# What Is the Giant Wall Gecko Having for Dinner? Conservation Genetics for Guiding Reserve Management in Cabo Verde

**DOI:** 10.3390/genes9120599

**Published:** 2018-12-03

**Authors:** Catarina Jesus Pinho, Bárbara Santos, Vanessa A. Mata, Mariana Seguro, Maria M. Romeiras, Ricardo Jorge Lopes, Raquel Vasconcelos

**Affiliations:** 1 CIBIO-InBIO, Centro de Investigação em Biodiversidade e Recursos Genéticos, Laboratório Associado da Universidade do Porto, Campus Agrário de Vairão, R. Padre Armando Quintas, 4485-661 Vairão, Portugal; cpinho541@gmail.com (C.J.P.); barbarasantosbio@gmail.com (B.S.); vanessamata@hotmail.com (V.A.M.); up201403024@fc.up.pt (M.S.); riclopes@me.com (R.J.L.); 2 Departamento de Biologia, Faculdade de Ciências da Universidade do Porto, R. Campo Alegre, 4169-007 Porto, Portugal; 3 Centre for Ecology, Evolution and Environmental Changes, Faculdade de Ciências Universidade de Lisboa, 1749-016 Lisboa, Portugal; mromeiras@yahoo.co.uk; 4 Linking Landscape, Environment, Agriculture and Food, Instituto Superior de Agronomia, Universidade de Lisboa, 1349-017 Lisboa, Portugal

**Keywords:** Desertas Islands, conservation, diet, metabarcoding, protected areas, *Tarentola gigas*

## Abstract

Knowledge on diet composition of a species is an important step to unveil its ecology and guide conservation actions. This is especially important for species that inhabit remote areas within biodiversity hotspots, with little information about their ecological roles. The emblematic giant wall gecko of Cabo Verde, *Tarentola gigas,* is restricted to the uninhabited Branco and Raso islets, and presents two subspecies. It is classified as Endangered, and locally Extinct on Santa Luzia Island; however, little information is known about its diet and behaviour. In this study, we identified the main plant, arthropods, and vertebrates consumed by both gecko subspecies using next generation sequencing (NGS) (metabarcoding of faecal pellets), and compared them with the species known to occur on Santa Luzia. Results showed that plants have a significant role as diet items and identified vertebrate and invertebrate taxa with higher taxonomic resolution than traditional methods. With this study, we now have data on the diet of both subspecies for evaluating the reintroduction of this threatened gecko on Santa Luzia as potentially successful, considering the generalist character of both populations. The information revealed by these ecological networks is important for the development of conservation plans by governmental authorities, and reinforces the essential and commonly neglected role of reptiles on island systems.

## 1. Introduction

Biodiversity is supported by an entangled network of interactions, and recognising this is crucial to guarantee the persistence of endemic and restricted-range taxa. The existing literature shows a clear bias towards certain taxonomic groups (e.g., for birds; see [1]), with reptiles typically receiving poor attention. Even though they are perceived as negligible, reptile species are important for several trophic ecological processes, especially on island ecosystems [2]. Gathering quantitative research on the subject is one of the solutions suggested to change this misleading paradigm [3].

The investigation of diet composition is one of the first steps to unveil species ecology and collect reliable data to guide conservation actions. Since detailed methods for faecal analysis were described, this technique has been widely used for determining the diet of many species, including reptiles [4]. This resulted in a large amount of information on biotic interactions of great importance to learn about their functional roles for the management of their habitats, which is especially valuable for threatened species. These entangled networks can only begin to be understood by a quantitative analysis of diets. However, several disadvantages of faecal analysis based on morphological identification of prey items have also been reported, as in these methods there is a tendency to underestimate the frequency occurrence and diversity [5], especially when only soft tissue has been ingested or when the prey is mainly soft-bodied. Also, this technique requires the knowledge of several experts to correctly identify the prey of different taxonomic groups, thus demanding a large expenditure of time [6].

To maximize the allocation of scarce resources for conservation efforts in the face of extensive anthropogenic threats, climate change, and accelerating extinction rates, the use of new and faster tools is needed [7]. This is especially relevant in developing countries within biodiversity hotspot areas. Next generation sequencing (NGS) metabarcoding is an advantageous alternative to classic approaches in the analysis of faecal pellets. This technique maximizes resolution, detection of rare events, and detection of soft, small, and invisible prey items. It can ultimately correct biases in ecological models, and is less reliant on taxonomic expertise [8]. Moreover, this method facilitates diet characterization for species that are difficult to observe in the act of eating, such as nocturnal reptile species [9], and that inhabit remote areas with an urgent need for conservation actions. Little is known about the dietary composition of geckos and their functional roles on island ecosystems. Some of the few existing studies were conducted based on direct observations and by using classic techniques to analyse faecal contents of the most widespread species, such as the moorish gecko *Tarentola mauritanica* (Linnaeus, 1758) [10,11] and the white-spotted gecko *Tarentola annularis* (Saint-Hilaire, 1827) [12,13].

The Cabo Verde Islands belong to the biogeographical region of Macaronesia and is a young developing country within the Mediterranean biodiversity hotspot. This archipelago is located in the Atlantic Ocean, approximately 500 km off the African coast, and comprises ten main islands and several islets, formed by a volcanic hotspot and never connected to the mainland [14]. Some of these islands and islets are presently uninhabited, such as the Santa Luzia Island and the Branco and Raso islets, and so are named the Desertas Islands. These are important breeding grounds for birds classified as Integral Nature Reserves since 1990 [15], and as a Marine Protected Area since 2003 [16].

This uninhabited tropical dry island group holds important endemic and highly-threatened species, some of them only occurring exclusively on these areas [17], yet are poorly studied due to their remoteness and harsh logistical constrains, such as the lack of potable water and need of special permits. These islands hold seven seabird and 11 terrestrial breeding bird species, and four threatened species of reptiles [18]. After birds, reptiles are the most important and the only terrestrial vertebrate group, and knowledge about their diet composition is also an indirect way of learning about the whole trophic network and the richness in biodiversity of these under-sampled areas.

An emblematic Cabo Verdean reptile is the Endangered giant wall gecko *Tarentola gigas* (Bocage, 1875), which is presently one of the largest geckonids in the world. This endemic species is predominantly nocturnal [19], oviparous [20], and actually restricted to Branco and Raso islets [21]. Subfossil evidence from owl pellets and other remains indicate that the species once inhabited the São Vicente and Santa Luzia islands, but disappeared from the diet of predators following human settlement and the introduction of mice and cats [22,23]. Little information is known about their population size (expected to fluctuate strongly due to fluctuating pressures on the populations of its prey species related with rainfall), diet, and behaviour in the two islets. Morphological analysis of the gecko’s faecal pellets and gut contents has already shown the presence of plants, invertebrates, fish scales, and seabird feathers [23]. Nevertheless, more information is needed on the ecology of the species; therefore, research is recommended for its conservation [24] and to investigate the possibility of its reintroduction on Santa Luzia [25]. Just recently, a restoration action plan was proposed for that island that includes the on-going removal of introduced mammal predators [26], enabling this conservation strategy to be set in action.

Concerning the functional role of *T. gigas*, the species is supposed to show strong trophic links with birds due to the scarcity of insects and other small prey on the islets [27]. The two subspecies were described as commensal of seabirds, as they normally inhabit their cliff-holes and burrows near the coast, although they can also be found under rocks inland [27]. The subspecies present on Raso, *T. gigas gigas* (Bocage, 1875), usually feeds on regurgitated food from several seabird species, but also on broken eggs and possibly the young of nesting birds [20,28]. It is probably the major natural predator of eggs of the Raso lark, *Alauda razae* (Alexander, 1898), a Critically Endangered bird now restricted to the Raso Islet [29], although that was not confirmed in a recent study based on morphological analyses of faecal pellets [30]. In this study, fishes were the most frequent item, followed by arthropods and plants. On Branco, where the Raso lark is absent, the subspecies *T. gigas brancoensis* (Schleich, 1984) presumably relies primarily on colonies of the Near Threatened endemic Cabo Verde shearwater, *Calonectris edwardsii* (Oustalet, 1883), though little information is available. Since the reintroduction of the Raso lark on Santa Luzia Island started in 2018 (with the release of around 25 birds and the first nestlings born in August), it is necessary to confirm and access the importance of the predation of this gecko on this bird species. Metabarcoding analysis can be really useful in this situation, as it provides, with less effort and time, a large and reliable amount of data, identifying multiple food items [8].

With this study, we intend to quantify the trophic interactions of the two subspecies, *T. gigas gigas* and *T. gigas brancoensis,* so that authorities can use this data to evaluate the reintroduction of this threatened gecko on Santa Luzia. For that, the main objective of this study is to identify the main plant, arthropod and bird species consumed by both subspecies of *T. gigas* using NGSmethods (metabarcoding of faecal pellets) and compare them with the species known to occur on Santa Luzia. The information revealed by these networks is of great importance to evidence the ecological role of reptiles in ecosystems, especially in islands where little is known, and to help in the development of conservation plans on these protected areas.

## 2. Materials and Methods

### 2.1. Study Area

**Figure 1:**
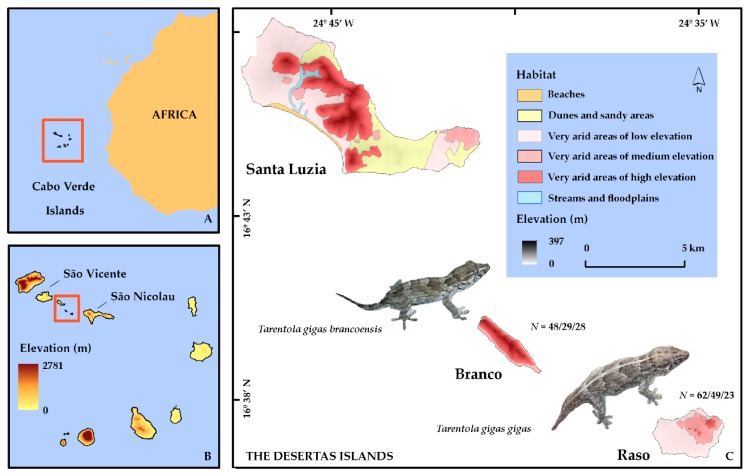
Studied area and taxa. Map of the Cabo Verde Islands, showing the (**A**) geographic location, (**B**) elevation, and (**C**) focusing on the Desertas group. The habitat types and the two studied subspecies are also represented, as well as the number of faecal samples (*N*) collected, extracted, and used in the analyses, respectively (Geographic Coordinate System, Datum WGS84).

This study took place on the Desertas Islands, composed of Santa Luzia Island and the Branco and Raso islets, located on the northwest alignment of the Cabo Verde Archipelago (16°48′ N, 24°47′ W and 16°36′ N, 24°34′ W; Figure 1A), flanked by the islands of São Vicente to the West and São Nicolau to the East (Figure 1B). The three islands have a total of 43.3 km^2^ of land area and present quite low elevations compared to the other islands of the archipelago. This group of islands is located at the border of the North African arid and semi-arid climatic regions, presenting a climate defined as being dry tropical Sahelian, predominantly represented by flat, very arid lowlands (Figure 1C), followed by very arid medium elevation areas, then beaches, dunes, and sandy areas, streams, and floodplains [31]. By means of the low elevation, the annual precipitation is among the lowest in Cabo Verde, which should be the primary limiting factor of the distribution of terrestrial biodiversity in the islands, leading to a low diversity of plant and insects in the area, mainly on Raso [18].

Santa Luzia presents a land area of approximately 35 km^2^ and has the highest elevation of the group, reaching 397 m. This island is very arid, yet there are more humid zones close to the river line, with hills, rocky plains, and sand dunes being the main landscapes. Branco Islet is the smallest of the group with a land area of approximately 3 km^2^. Mountainous (2 km^2^) and medium-elevation (1 km^2^) arid areas dominate the islet’s landscape [32]. The islet is of difficult access due to the roughness of the sea, lack of safe natural ports, and steepness (there is only a minor area of plane ground of about 400 × 200 m). Raso Islet has a land area less than 6 km^2^ and, in contrast with Branco, is almost flat in all its extent. This islet is fundamentally characterized by plains and low-altitude arid zones (Figure 1C) with patches of grassy vegetation.

### 2.2. Study Species

The Cabo Verde giant wall gecko is the largest gecko in Cabo Verde, reaching a maximum of 155 mm from snout to vent [21]. It was classified as Endangered in the International Union for Conservation of Nature (IUCN) Red List of Threatened Species, mainly due to its reduced distribution and exploitation of its prey species [24]. The subspecies *T. gigas gigas* inhabits the Raso islet and differs morphologically from the subspecies of the Branco islet, *T. gigas brancoensis*, by its longer snout, larger number of scales at mid-body, and by the proportion between the width and length of the fourth toe, normally lower than 1:5 [21,33,34]. As mentioned above, both subspecies are nocturnal, oviparous, most ground-dwelling, and use rocks and seabird’s burrows as diurnal refuges [21,24,33,34].

### 2.3. Sampling

Sampling on Raso took place from September to December 2016 and on Branco during September 2017. Based on precipitation data from São Pedro (São Vicente) weather station (https://cv.freemeteo.com), these months belonged to the wet season. Several different sites of the islets were sampled with the purpose of embracing all possible habitats that could provide different food resources for the reptiles, overlapping with occurrence sites of several bird species. We collected 62 specimens in Raso and 48 in Branco (Figure 1C). All of them were captured by hand, and received a belly massage in order to release the fresh pellets, which were preserved in tubes with 96% ethanol. The individuals were also sexed based on the absence or presence of cloacal pouches [21], measured (snout–vent length (SVL) to the nearest mm), and a sample of the tip of the tail was collected before releasing each animal. Each sample was geolocated using a GPS device, and photos were taken to confirm the data in case of uncertainties (e.g., sexing). Sampling and protocols were approved by “Direcção Geral do Ambiente” (DNA), Cabo Verde (no 58/2017).

Samples of invertebrates, vertebrates, and plants were collected from Santa Luzia, Raso, and Branco in order to build a DNA reference collection of possible food items. For the collection of invertebrates, pitfalls were placed on each island in two areas of the islands (sandy and compact soil). Replicas were set on a different location with the same soil type on each island to gather a representative sample of the island invertebrate biodiversity. Specimens were separated in different high-level taxonomic groups based on morphological identification, and photographed with a camera assembled on a magnifying lens for morphological identification at higher taxonomical resolution by experts. Vertebrate samples were collected in the field from dead animals or traces of their presence, such as feathers and eggshells. Samples of leaves and flowers were collected across the islands, and pictures were also taken in the field to allow for morphological taxonomic assessment by experts.

### 2.4. DNA Extraction and Amplification

The collected *T. gigas* pellets were completely dehydrated in an incubator at 50 °C in order to remove all traces of ethanol. Then, DNA was extracted using the Stool DNA Isolation Kit (Norgen Biotek Corp., Thorold, ON, Canada), following the manufacturer’s instructions. Two DNA elutions were obtained in a total volume of 50 µL each and were frozen at −20 °C until DNA amplification.

The plant reference library was constructed by extracting DNA from leaves and flowers collected in the islands, using the DNeasy Plant Mini Kit (Qiagen, Crawley, UK) following some alterations according to [35]. Invertebrate DNA extraction was carried out from a leg or wing sample of each different Operational Taxonomic Unit (OTU) identified by the experts, using saline extraction methods [36]. This protocol was also used for the DNA extraction of vertebrates.

Three different DNA fragments were chosen to identify the distinct prey groups (plants, invertebrates, and vertebrates) that presumably compose the diet of the study species. For plants, the g/h primers were used targeting the short P6-loop of chloroplast *trnL* (UAA) (see Supplementary Table S1, [37]) intron, and for invertebrates, a modified version of the IN16STK-1F/IN16STK-1R primers was used, targeting the mitochondrial 16S rRNA (Supplementary Table S1). The 16S primers were specifically designed to amplify the insect diet of lizards while avoiding the amplification of the lizards, while *trnL* has been extensively used in both environmental DNA (eDNA) assessments and diet studies. For the amplification of vertebrate DNA, the 12sv5F and 12Ssv5R primers targeting the V5-loop fragment of the mitochondrial 12S gene (Supplementary Table S1) were used. This marker has been shown to have great resolution power for genus and species identification across numerous vertebrate taxa [38]. To avoid the amplification of *T. gigas* DNA, a blocking primer (5′-CCCCACTATGCTCAACCGTTAACAAAG(C3 spacer)-3′) was used. This primer was designed by building an alignment with available 12S sequences of this species, as well as of birds and fishes known to occur in Cabo Verde or of taxonomically related species. We further modified all primers in order to contain Illumina adaptors and a 5 bp individual identification barcode to allow for individual identification of each sample.

For the *trnL* and 16S markers, PCR reactions were carried out in volumes of 25 µL, comprising 10.4 µL of QIAGEN Multiplex PCR Master Mix (Quiagen, Crawley, UK) 0.4 µL of each 10µM primer, 10.8 µL of ultra-pure water, and 3 µL of DNA extract. Cycling conditions used an initial denaturing at 95 °C for 15 min, followed by 39 cycles of denaturing at 95 °C for 30 s, annealing at 45 °C and 52 °C, respectively, for 30 s and extension at 72 °C for 30 s, with a final extension at 72 °C for 10 min. For the 12S marker, PCR reactions were carried out in volumes of 25 µL, comprising 10.4 µL of QIAGEN Multiplex PCR Master Mix, 0.4 µL of each 10 µM primer, 8 µL of 10 µM blocking primer, 2.8 µL of ultra-pure water, and 3 µL of DNA extract. Cycling conditions used an initial denaturing at 95 °C for 15 min, followed by 39 cycles of denaturing at 95 °C for 30 s, annealing at 48 °C for 30 s, and extension at 72 °C for 30 s, with a final extension at 72 °C for 10 min.

Reference collection plant samples were amplified for the chloroplast *trnL* (UAA) using primer “e” and “f” [39]. The PCR reactions were carried out in volumes of 25 µL, comprising 4 µL of QIAGEN Multiplex PCR Master Mix, 1 µL of each 10 µM primer, 16.4 µL of ultrapure water, 0.5 µL of bovine serum albumin (BSA; 20 mg/mL), and 3 µL of DNA extract. Cycling conditions used an initial denaturing at 94 °C for 10 min, followed by 30 cycles of denaturing at 94 °C for 1 min, annealing at 50 °C for 3 min, and extension at 72 °C for 1 min, with a final extension at 72 °C for 8 min. Invertebrate and vertebrate DNA for the reference collection was amplified for the same markers stated before for these groups, to allow the match with the dietary sequences. The DNA from invertebrates was amplified for 16S using the same PCR conditions; however, these samples were also sequenced for the standard cytochrome oxidase I (COI) barcode fragment using LCO1490/HC02198 following the PCR conditions as described in [40], allowing, in this way, to confirm dubious taxonomic assignments. PCR reactions for vertebrate DNA were carried out in volumes of 25 µL, comprising 10.4 µL of QIAGEN Multiplex PCR Master Mix, 0.4 µL of each 10 µM primer, 11.8 µL of ultra-pure water, and 2 µL of DNA extract, following the same cycling conditions described before for this marker.

### 2.5. Library Preparation

Succeeding amplification, library preparation was carried out following the Illumina MiSeq protocol 16S Metagenomic Sequencing Library Preparation [41]. Before sequencing, PCR products were cleaned using Agencourt AMPure XP beads (Beckman Coulter, Brea, CA, USA) to remove free primers and primer dimers, following two cleaning steps with ethanol and a final dilution using 10nM Tris. The purified products were quantified using the NanoDrop 2000 spectrophotometer (Thermo Scientific, Waltham, MA, USA), and subsequently normalized to 10 ng/μL. Samples amplified with different barcodes were pooled together. Afterwards, an indexing PCR was performed for the incorporation of the Illumina-compatible indexing primers to each pool using the Nextera XT Kit (Illumina, San Diego, CA, USA), allowing individual identification of each amplified product. The PCR reactions and cycling conditions were similar to the ones of the first PCR, except that only eight cycles of denaturing, annealing, and extension were done, with annealing at 55 °C. The indexed PCR products were again cleaned, quantified, and pooled at equimolar concentrations (15 nM). The final pool was quantified by qPCR (KAPA Library Quant Kit qPCR Mix, Bio-Rad iCycler, Hercules, CA, USA), diluted to 4 nM, and run in a MiSeq sequencer (Illumina) using a 2 × 150 bp MiSeq Reagent Kit (Illumina, San Diego, CA, USA) for an expected average of 12,000 paired-end reads per sample.

The reference collection samples amplified for COI and *trnL* markers were sequenced using Sanger sequencing.

### 2.6. Bioinformatics

The software package OBItools (https://git.metabarcoding.org/obitools/obitools) was used for general sequence processing (as described in [42]). Forward and reverse sequences were aligned (command illuminapairedend) and discarded when overlapping quality was less than 40. Reads were then assigned to samples and primers and barcodes were removed (command ngsfilter), allowing a total of four mismatches to the expected primer sequence. The reads were then collapsed into unique haplotypes. Singletons (haplotypes with only one read) and the potentially erroneous haplotypes resultant from PCR errors were deleted (command obiclean) by removing haplotypes that differed by 1 bp from the most abundant haplotypes. This way, any “A” haplotype differing one base-pair from a “B” haplotype, with an absolute read count lower than “B”, and that was not found without the presence of “B” in any sample, was removed. After this step, the samples with less than 100 reads in total were considered to have failed and removed. For the remaining ones, haplotypes representing less than 1% of the total number were removed from each sample [42].

Haplotypes were identified by comparing the final set against the GenBank online database (https://www.ncbi.nlm.nih.gov/), as well as the obtained reference samples. The sequences with less than 90% of similarity between known species were classified only to class level, the ones with similarity between 90 and 95% were classified to the family level, and sequences presenting more than 95% of similarity between known species were classified to species or genus level. The obtained results were also compared with Cabo Verde databases referred to in the literature [17] and other databases for birds (https://avibase.bsc-eoc.org), marine species (http://www.marinespecies.org/), and the encyclopedia of life (http://eol.org). When the same haplotype matched more than one species or genus with similar probabilities, there were only considered species or genera known to occur in Cabo Verde. After identifying all haplotypes, the ones with a high probability of arising from lab contaminations were discarded.

### 2.7. Data Analysis

Frequencies of occurrence of plants, invertebrates, and vertebrates (fishes, reptiles, and birds) were estimated for both diets. Overlap on the occurrence of the plants, invertebrates, and vertebrates was visualized with Euler proportional elliptic diagrams, using the Euler command from the package eulerr [43] of the statistical environment R 3.4.1., to check the possibility of secondary consumption and the generalist/specialist character of the individuals. Differences of frequencies of each group between diets were compared using chi-square tests in the same statistical environment. They were also compared with previous published results [30].

In order to assess if there were differences in prey species richness between the two islets measured in Molecular Operational Taxonomic Units (MOTUs), we compared MOTU richness using a chi-square comparison and calculated asymptotic MOTU richness and 95% confidence intervals with an endpoint of 1000 samples, using command iNEXT from the INEXT package [44], using R 3.4.1.

A permutational multivariate analysis of variance (PERMANOVA) was carried out using the vegan package (function ADONIS) with the aim of comparing diet composition between the two subspecies of each islet [45]. A matrix of the presence of each MOTU in all samples was made. For the invertebrates, due to the lack of taxonomic resolution, MOTUs were grouped into families. A dissimilarity matrix was calculated using the Jaccard measure, due to the binary (presence/absence) nature of our data. A homogeneity of dispersion test (PERMDISP) was also carried out in order to assure the significance of the PERMANOVA test, as it assumes an equal dispersion of values across the different groups. Afterwards, a similarity percentage analysis was performed, also using the vegan package (function simper), to infer the contribution of each prey to the differentiation between diets. Also, the Czekanowski niche overlap index was calculated to understand the niche overlap between the two subspecies’ diets using “command czekanowski” from the EcoSimR package [46], using R 3.4.1.

## 3. Results

A total of 110 faecal samples were collected (Raso = 62; Branco = 48) of which 78 samples showed clear signs of amplification and were therefore sequenced (Raso = 49; Branco = 29). After all the analytical and bioinformatics procedures, our final dataset comprised 51 samples (Raso = 23; Branco = 28).

Overall, we identified 139 prey items of 11 taxonomic classes, from plants to birds (Supplementary Table S2). Plants were distributed among three classes, 17 orders, and 21 families (Zygophyllaceae occurred more frequently). Invertebrates from five classes, 13 orders, and 42 families were detected, with higher frequencies of Noctuidae (Lepidoptera) and Culicidae (Diptera). Vertebrates were identified from three classes, seven orders, and 12 families. Some families were exclusively detected in one subspecies diet. For example, Tenebrionidae invertebrates were only present in the *T. gigas brancoensis* subspecies, while Aizoaceae plants were only found in the *T. gigas gigas* subspecies.

**Figure 2:**
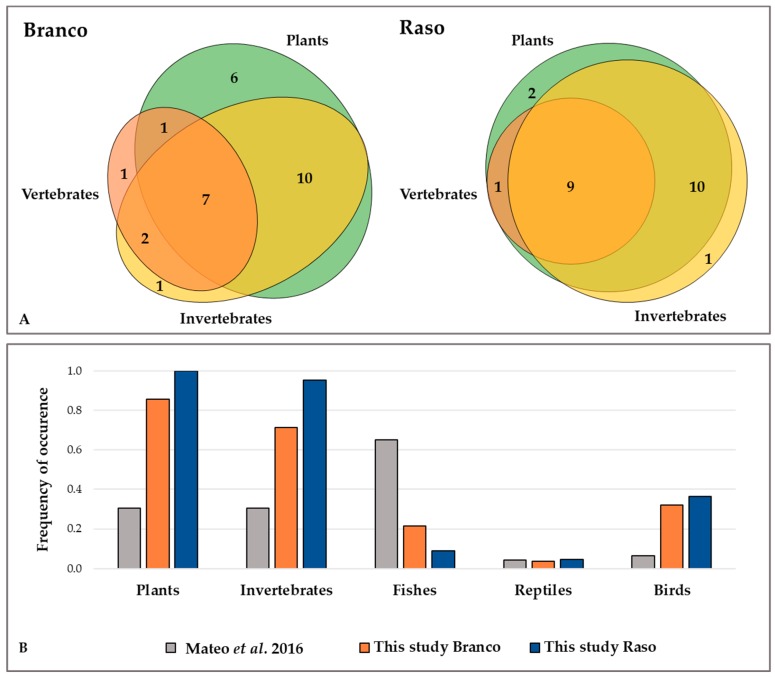
Metabarcoding results for each subspecies and comparison with classic methods. (**A**) Euler diagrams showing the occurrence and overlap of the three main prey groups (plants in green, invertebrates in yellow, and vertebrates in orange) in the faecal samples from the Branco and Raso islets, respectively. (**B**) Frequencies of occurrence of plants, invertebrates, and vertebrates (fishes, reptiles, and birds) in the faecal samples from the Branco (orange) and Raso islets (blue). The results from a previous study [30] are also shown for comparison (grey).

The occurrence of the three taxonomic groups (plants, invertebrates, and vertebrates) in both diets was very similar, with a high overlap of groups and very few samples with just one taxonomic group detected (Figure 2A). The overlap was higher between plants and invertebrates (plants and invertebrates: Raso = 54%, Branco = 35%) than between the other combinations of groups. The number of samples with an overlap between plants and invertebrates was also higher than the number of samples with an overlap of all three groups (plants, invertebrates, and vertebrates: Raso = 37%, Branco = 25%). In both diets, plants and invertebrates were the most frequent groups, followed by birds and reptiles (Figure 2B). On Raso, the frequency of all groups was higher (plants: *Χ*^2^ (1) = 1.751, *p* = 0.186; invertebrates: *Χ*^2^ (1) = 3.328, *p* = 0.068, birds: *Χ*^2^ (1) = 0.000, *p* = 0.990; reptiles: *Χ*^2^ (1) = 3.59 × e^−31^, *p* = 1.000) with the exception of fishes, that were more frequent in Branco samples (*Χ*^2^ (1) = 0.628, *p* = 0.428), yet these differences were not significant. In comparison with previously published results [30], the number of occurrences was always higher for all taxonomic groups, with the exception of fishes. Although plants had a higher occurrence, invertebrates showed a higher diversity of MOTUs (Supplementary Table S2).

**Figure 3:**
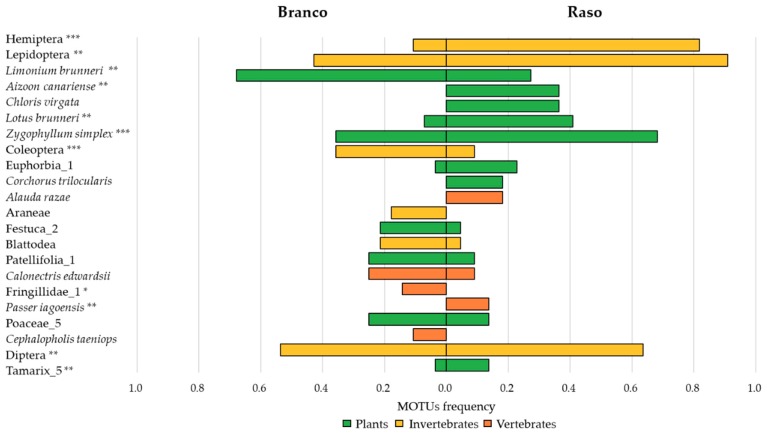
Results of the similarity percentage analysis. Frequency of occurrence of Molecular Operational Taxonomic Units (MOTUs) with the highest contribution to differences between the diets of *Tarentola gigas* in both islets. Magnitude of significance levels shown with asterisks: *** *p* < 0.001; ** *p* < 0.01; * *p* < 0.05.

Species richness was similar between diets (Raso = 84, Branco = 95, *Χ*^2^ (1) = 0.032, *p* = 0.932). The extrapolated species richness in Branco was higher, but with high overlap on the lower limit of the 95% confidence interval (Raso = 208 ± 53.8 (139–364); Branco = 249 ± 60.7 (168–419)). There were significant differences in the MOTU composition between diets (Supplementary Table S3), and no effect of data dispersion on the results (Supplementary Table S4). This was corroborated by the low overlap between diet MOTUs (Czekanowski index = 0.31). Hemiptera and Lepidoptera were the MOTUs that contributed the most for the differences between diets, followed by a diverse set of MOTUs belonging to plants and birds (Figure 3).

## 4. Discussion

Our study reveals the first DNA-based data on the diet of the two subspecies of the Endangered and endemic Cabo Verdean *T. gigas*. In our study, plants and invertebrates were the most frequent groups, followed by birds. Even though our results cannot be compared in a straightforward way to the ones based purely on the morphological examination previously published [30], since their sampling took place in the dry season and our sampling in the wet season, both confirm that the study species has a generalist diet, feeding on plants, invertebrates, and vertebrates. However, in our study, the number of occurrences is always higher for all taxonomic groups, apart from fishes, where the detection was higher in the classic study, and reptiles that were detected in similar proportions (Figure 2B). The differences in the incidence of fishes can be justified by a sampling bias in the previous works [30], considering that all their samples were collected near seabird colonies where the fish remains are common, whereas our sampling on Raso was more widespread.

Plants and invertebrates presented the highest differences between the two studies (Figure 2B). This was expected, as with morphological examination there is a tendency to underestimate prey incidence, considering that only partially digested or items with non-digestible parts can be detected [47,48], whereas with metabarcoding it is possible to detect small, soft, and invisible items. Moreover, the samples used in the previous work were possibly dated from 1999 [30] and could be in a more degraded state, making prey identification difficult. Nonetheless, plant items occurred in a higher frequency in the diet of *T. gigas* than what was previously thought. In a recent study on the diet of *Tarentola raziana*, similar results were revealed to this syntopic species [49]. We found 20 plant families consumed by this gecko, while the previous report only stated the presence of Poacea, and occasional observations of Schleich [20,50] did not mention plants at all. Additionally, we could reach a higher taxonomic resolution than earlier studies. In all three taxonomic groups, we were able to identify some items to the species level (Supplementary Table S2), whereas in the previous work, only *Sula leucogaster* and *C. edwardsii* were identified at the same taxonomical resolution [30]. For invertebrates, only Coleoptera, Diptera, Orthoptera, Hymenoptera, and Mantodea orders were formerly detected [20,30,50]. Even though we were unable to detect Mantodea, we identified nine additional orders formerly undescribed for the *T. gigas* diet. Also, previous authors reported one case of cannibalism and one ingestion of *T. raziana* [30], which could not be confirmed by our study as we used a blocking primer to prevent DNA amplification of *Tarentola*. However, we identified the occurrence of *Chioninia stangeri* (Gray, 1845) which can be an indicator of the predation on other reptiles (dead or alive). There is previous evidence of predation by larger species of lizards, as is the case of Lehrs’ lizard, *Gallotia caesaris* (Lehrs, 1914), on smaller ones [51], so this is expected to be even more common on other islands’ systems where the resources are more limited. Apart from the impossibility of detecting cannibalism, metabarcoding dietary studies are somehow affected by the deficiency of DNA reference sequences and the Linnean shortfall [8]. We started the construction of a reference database for collections of the flora and fauna for our system that was very helpful in correctly identifying some diet items; however, higher resolution of taxa identification would be possible if there were up-to-date species checklists and more sequenced taxa of these poorly studied islands. Some other limitations of this approach is the possibility of false inferences due to contaminations, therefore a careful interpretation of doubtful taxa was carried out, probably discarding true positives. Finally, we needed to have in consideration that this method only provides the taxa occurrences in the samples and not their relative abundances [8]. Nevertheless, as the technological procedures evolve in accelerated rates, these and other metabarcoding issues discussed above are being solved. It is expected that these tools will continue to improve, reducing the costs and providing high-quality data to better guide conservation planning of excellency [7].The Euler diagrams show high overlap on the detection of taxonomic groups (plants, invertebrates, and vertebrates) for both populations, with very few samples with just one taxonomic group detected, reinforcing the evidence for the generalist diet of the species at the individual level. The overlap between plants and invertebrates was also higher than the number of samples with an overlap of all three groups. This may be explained by the secondary consumption of plants when invertebrates are consumed [52], and if this is the case, we may be detecting plant DNA consumed by the arthropod species, leading to an overestimation of the plant items consumed. On the other hand, ten of the samples contained only plants. In many cases, islands lizards have fewer terrestrial predators and are capable of reaching higher densities. This, along with a lower availability of insects for preying and the arid conditions of the study area, may have favoured the importance of plant items in their diet. Additionally, higher plant-matter ingestion is associated with species with large body and gut sizes [53], characteristics owned by *T. gigas* specimens (personal observation). Herbivory in lizards is also associated with arid, warmer areas and ecosystems with few competitors and predators [54], such as this one. Indirectly, insular lizards may then have an important role in seed dispersal and pollination in these systems [55], which could be the case of our study species. In fact, we found several geckos with their snouts covered with pollen during sampling on Branco, and so this deserves further study. Finally, both previously published works [20,30] found small stones in the pellets, known to be swallowed to help plant digestion [56].

We found a higher observed and extrapolated MOTU richness in Branco. However, the difference between both diets has a small ecological magnitude and may be due to the reduced sample size, since the confidence intervals partially overlap. Despite the more homogeneous sampling effort, as the Branco samples were collected only in five days of the same month and in a restricted area due to the roughness of the ground, we may argue that we could find a higher diversity in Branco pellets if we sampled during a longer period of time and in a wider range of habitats. This would be expected taking into account that Branco presents a higher altitudinal gradient than Raso, which is nearly flat in its extent. Branco consequently presents higher humidity levels, thus embracing a greater variety of niches that can hold a higher diversity of plants and invertebrates.

More explicitly focusing on the MOTUs that revealed a higher contribution for the differences between the diets of the two gecko populations, some invertebrates contributed to the higher differences in the two diets, namely the Hemiptera and Lepidoptera species. Unfortunately, most MOTUs were not possible to be identified to the highest taxonomic resolution, providing little information to infer whether one of the two populations particularly relies on a certain arthropod group. Barely any information also exists on the status and distribution of invertebrates on these islands; therefore, we were not able to compare which gecko population would be better for reintroduction, based solely on the incidence of this group. It is necessary to improve the reference collections and catalogues of the diverse species of arthropods which inhabit the Desertas islands to make further conclusions.

Several plants also appear to be important—some are exotic, such as the *Chloris virgata* Swartz, while others are native, such as the *Zygophyllum simplex* L., and still others are endemic of Cabo Verde Archipelago, such as the *Limonium brunneri* (Webb) Kuntze which is consumed more frequently by the Branco population. This may happen due to a higher availability of this taxon on Branco, or a preference for *T. g. brancoensis*. Given the generalist character of this gecko diet, the first hypothesis is more likely. This species occurs both on Branco and Raso, as well as on Santa Luzia. It is classified as Critically Endangered due to its restricted distribution, and its population seems to be decreasing on Santa Luzia reserve [57]. The reintroduction of *T. gigas* on Santa Luzia could favour pollination and/or the dispersal of seeds, depending on the parts of the plant that are ingested. It is necessary to improve data on this to understand the extent of this service to make further conclusions. Concerning vertebrate MOTUs, we found that some bird species are important in the Raso subspecies diet, but not for the Branco one. This is the case of the Endangered species *A. razae* and other Passeriformes with low populations sizes, such as *Passer iagoensis* (Gould, 1837). This is an expected result, since these prey items do not occur in Branco (*A. razae*) or their breeding is unknown (*P. iagoensis*). However, we confirmed that the Branco population preys with more frequency on the Near Threatened *C. edwardsii*. A strong commensal link of *T. gigas brancoensis* with these seabirds could also explain the higher frequencies of fish found on Branco.

Considering the overall obtained data, the most important fact is that the diet of this gecko, in both islands is rather generalist. This means that differences in the diet between sites may be more due to species availability rather than population differences in trophic ecology. In the perspective of the reintroduction of this gecko on Santa Luzia, our data needs to be interpreted as valuable for the integration with other kinds of data. Concerning the survival of reintroduced geckos due to diet requirements, we consider that both populations could be reintroduced on Santa Luzia. The Branco population seems to have a wider range of diet items, and their acclimatisation on Santa Luzia could be easier than for the Raso population, which, due to the more homogenous range of habitats, seems to have a less diverse diet. Also, *T. gigas brancoensis* is probably the subspecies more genetically closer to the extinct population of Santa Luzia (based on geographic distances [21] and the age of the islands [58]). Branco is also the geographically closest islet to Santa Luzia Island. This would be economically more advantageous and safer, due to the roughness of the sea. On the other hand, disembarking on Raso is relatively easier and fieldworkers have more temporary conditions to perform field work, despite being more distant. Concerning the success of the ongoing translocation of *A. razae*, and depending on the overlap of the distributions of both species, the introduction of a known predator could have a negative impact on the survival of the bird nestlings. Since the population of Branco is not used to preying on Raso larks, it could be naïve to prey on this bird for a first stage, and a better choice for successful acclimatisation of the birds. However, due to the generalist character of both diets, this is just one scenario among many, and it is probably advisable to obtain more data to model the impact of another predator on the viability and growth of this new *A. razae* population before any action is taken. Overall, an evaluation of the best population source would benefit from the inclusion of data about the genetic diversity and similarity between subspecies (ongoing), the densities of each population, and a careful analysis of the cost–benefits of each option.

## 5. Conclusions

Our results revealed that *T. gigas* has a generalist diet that encompasses most of the diversity of resources found in both islands, from plants to birds. In the future, it would be interesting to understand the importance of this gecko in connecting the marine and terrestrial ecosystems by recycling nutrients (e.g., ingestion of regurgitated fishes by birds on Branco), whether they have a significant phytosanitary effect that keeps bird populations free of diseases, or to what extent the species provides ecological services to the maintenance of threatened plant species. All these hypotheses require future research, and for that we need to expand our knowledge on the plants and invertebrates present on Santa Luzia, by completing our reference collection and improving the list of described species for the island. This would allow us to analyse and obtain more insights to understand the ecological interaction of *T. gigas* with plant and invertebrate species.

## Supplementary Materials

The following are available online at http://www.mdpi.com/2073-4425/9/12/599/s1, Table S1: Primer sequences forward and reverse used in this study, Table S2: List of the identified MOTUs to the maximum resolution obtained, with the respective occurrence in the samples of *Tarentola gigas brancoensis* (*Tgb*) and *Tarentola gigas gigas* (*Tgg*), Table S3: PerMANOVA results of island effect in the diet of the two subspecies of *Tarentola gigas*., Table S4: PerMADISP test results.
