# Supplementary material for: What Is the Giant Wall Gecko Having for Dinner? Conservation Genetics for Guiding Reserve Management in Cabo Verde
Source: Genes (Basel). 2018 Dec 3;9(12):599. doi: 10.3390/genes9120599 (PMC6315674; doi:10.3390/genes9120599)
Supplement: Supplementary file 1 [file genes-09-00599-s001.pdf]

Supplementary Materials

Table S1. Primer sequences forward and reverse used in this study

Amplified region	Fragment	Primer sets	Primer sequence	Reference
V5-loop (12s)	73–110	12sv5F	TAGAACAGGCTCCTCTAG	[38]
		12sv5R	TTAGATACCCCACTATGC	
16S rRNA	~ 110	IN16STK-1F-mod	TRAACTCAGATCATGTAA	This manuscript (based on[9])
		IN16STK-1R-mod	TTAGGGATAACAGCGTWA	
P6-loop (chloroplast trnL)	10–143	g_F	GGGCAATCCTGAGCCAA	[37]
		h_R	CCATTGAGTCTCTGCACCTATC	

Table S2. List of the identified MOTUs to the maximum resolution obtained, with the respective occurrence in the samples of *Tarentola gigas brancoensis* (*Tgb*) and *Tarentola gigas gigas* (*Tgg*).

Phyllum	Order	Family	Final ID	*Tgb*	*Tgg*
**Tracheophyta**					
**Brassicales**	Capparales	Brassicaceae	Lobularia_1	6	3
**Liliopsida**	Poales	Poaceae	*Chloris virgata*	0	8
			Festuca_1	0	1
			Festuca_2	6	1
			Poaceae_2	2	2
			Poaceae_5	7	3
			Poaceae_7	0	2
**Magnoliopsida**	Apiales	Apiaceae	Apiaceae_2	2	1
	Aquifoliales	Aquifoliaceae	*Ilex_1*	1	0
	Asterales	Asteraceae	Asteraceae_1	0	1
			Asteraceae_2	1	0
	Caryophyllales	Aizoaceae	*Aizoon canariense*	0	8
		Plumbaginaceae	*Limonium brunneri*	20	6
		Tamaricaceae	*Tamarix_5*	1	3
	Caryophyllales	Amaranthaceae	*Chenopodium murale*	5	2
			Chenopodium_2	1	0
			Patellifolia_1	7	2
			Patellifolia_2	1	0
	Cucurbitales	Cucurbitaceae	Cucurbitaceae_1	1	1
	Fabales	Fabaceae	Fabaceae_3	1	0
			Fabaceae_4	1	0
			Fabaceae_5	1	0
			Lotus brunneri	2	9
	Lamiales	Lamiacea	*Lavandula coronopifolia*	0	1
	Laurales	Lauraceae	Lauraceae_1	2	0
	Malpighiales	Euphorbiaceae	*Euphorbia_1*	1	5
	Malvales	Malvaceae	*Corchorus trilocularis*	0	4
	Myrtales	Lythraceae	Lythraceae_1	1	0
	Rosales	Rosaceae	Rosaceae_3	1	0
	Sapindales	Anacardiaceae	*Rhus_1*	1	0
	Solanales	Convolvulaceae	Convolvulaceae_1	1	1
		Solanaceae	Solanaceae_3	1	0
	Zygophyllales	Zygophyllaceae	*Tribulus cistoides*	4	3

Phyllum	Order	Family	Final ID	*Tgb*	*Tgg*
			Zygophyllum simplex	10	15
**Arthropoda**					
**Arachnida**	Araneae	Gnaphosidae	*Drassodes_2*	1	0
		Oecobiidae	*Uroctea_1*	1	0
		Philodromidae	*Thanatus vulgaris*	1	0
		Selenopidae	Selenopidae_1	2	0
**Chilopoda**	UNK	UNK	Chilopoda_2	0	1
**Collembola**	UNK	UNK	Collembola_1	0	1
**Insecta**	UNK	UNK	Insecta_16	0	1
	Blattodea	UNK	Blattodea_4	1	1
			Blattodea_5	1	0
			Blattodea_6	6	0
	Coleoptera	UNK	Coleoptera_10	1	0
		Carabidae	Carabidae_3	0	1
		Cerambycidae	Cerambycidae_1	0	1
		Coccinellidae	Coccinellidae_1	1	0
		Dermestidae	*Dermestes_1*	1	0
		Scarabaeidae	Scarabaeidae_1	1	0
		Staphylinidae	Staphylinidae_4	3	0
		Tenebrionidae	Tenebrionidae_3	1	0
			Tenebrionidae_4	7	0
		Tetratomidae	Tetratomidae_1	1	0
	Diptera	UNK	Diptera_6	3	6
		Agromyzidae	Agromyzidae_1	0	1
		Cecidomyiidae	*Mayetiola destructor*	1	0
			Mayetiola_1	1	0
		Ceratopogonidae	*Culicoides_1*	1	0
		Chironomidae	*Chironomidae_1*	0	1
			Chironomus tepperi	3	3
			Cricotopus_1	6	1
		Culicidae	*Aedes_1*	0	1
			Culicidae_3	10	6
		Drosophilidae	Drosophilidae_1	2	0
		Psychodidae	Psychodidae_1	3	0
		Sarcophagidae	Sarcophagidae_1	1	0
			Wohlfahrtia_1	1	1
		Sciaridae	Sciaridae_1	2	1
	Hemiptera	UNK	Hemiptera_2	1	3
		Acanthosomatidae	Acanthosomatidae_1	0	1
		Aphididae	*Aphis_1*	1	0
		Cicadellidae	Cicadellidae_1	0	5
			Orosius_1	1	1
		Lygaeidae	Lygaeidae_1	0	1
			Nysius_1	0	1
			Nysius_2	0	1
		Nabidae	Nabidae_2	0	14
		Pentatomidae	*Halyomorpha halys*	0	1
			Pentatomidae_1	0	1
			Pentatomidae_3	0	6
		Pteromalidae	Pteromalidae_2	0	1
		Apidae	Apidae_3	1	0
	Hymenoptera	Formicidae	*Solenopsis_1*	1	1
		Thynnidae	Tiphiidae_2	1	0
		Tiphiidae	Tiphiidae_3	2	0
	Lepidoptera	UNK	Lepidoptera_12	0	1

Phyllum	Order	Family	Final ID	*Tgb*	*Tgg*
			Lepidoptera_17	0	1
			Lepidoptera_10	1	0
			Lepidoptera_16	1	0
			Lepidoptera_23	0	1
			Lepidoptera_24	4	1
			Lepidoptera_26	0	1
			Lepidoptera_30	1	0
			Lepidoptera_4	1	0
		Crambidae	Crambidae_1	0	1
			Crambidae_2	0	2
			Nomophila noctuella	0	1
			Tegostoma_1	1	0
			Tegostoma_2	1	0
			Tegostoma_3	2	1
		Noctuidae	*Acraprex_1*	0	1
			Agrotis_1	2	14
			Agrotis_2	0	6
			Agrotis_4	3	2
			Noctuidae_2	1	1
			Noctuidae_3	0	1
			Noctuidae_5	0	1
			Noctuidae_6	0	14
		Pieridae	Pieridae	0	2
	Neuroptera	UNK	Neuroptera_1	0	1
		Myrmeleontidae	Myrmeleontidae_1	2	0
	Odonata	Calopterygidae	Calopterygidae_1	2	0
		Coenagrionidae	Coenagrionidae_1	1	0
		Libellulidae	Libellulidae_1	0	1
	Orthoptera	Acrididae	Acrididae_2	1	0
			Acrididae_5	6	2
			Schistocerca_1	0	2
		Gryllidae	Gryllidae_1	1	0
	Zygentoma	UNK	Zygentoma_1	0	1
		Lepismatidae	*Heterolepisma_10*	1	0
			Heterolepisma_11	1	0
			Heterolepisma_3	1	0
			Lepismatidae_8	0	1
			Thermobia domestica	1	0
**Malacostraca**	Decapoda	UNK	Decapoda_1	1	1
	Isopoda	UNK	Isopoda_1	0	1
**Chordata**					
**Actinopterygii**	Beloniformes	Belonidae	*Tylosurus _1*	0	1
		Hemiramphidae	*Hemiramphus*	1	0
	Carangiformes	Carangidae	*Trachinotus ovatus*	0	1
	Perciformes	Acanthuridae	*Acanthurus_1*	1	0
		Epinephelidae	*Cephalopholis taeniops*	3	0
		Scaridae	*Sparisoma_1*	1	0
	Salmoniformes	Salmonidae	*Oncorhynchus_1*	1	0
**Aves**	Passeriformes	Alaudidae	*Alauda razae*	0	4
		Fringillidae	Fringillidae_1	4	0
		Passeridae	*Passer iagoensis*	0	3
	Procellariiformes	Procellariidae	*Calonectris edwardsii*	7	2
**Reptilia**	Squamata	Scincidae	*Chioninia stangeri*	1	1

Table S3. PerMANOVA results of island effect in the diet of the two subspecies of *Tarentola gigas*. d.f. stands for degrees of freedom, SS for sum of squares, and MS for mean of squares.

Variable	d.f.	SS	MS	F Model	R2	Pr (>F)
Islet	1	1.8438	1.84383	5.5849	0.10423	0.001
Residuals	48	15.8470	0.33015		0.89577	
Total	49	17.6908			1.00000	

Table S4. PerMADISP test results. d.f. stands for degrees of freedom, SS for sum of squares, and MS for mean of squares.

	d.f.	SS	MS	F Model	Pr (>F)
Groups	1	0.11085	0.11085	11.186	0.001606
Residuals	48	0.47568	0.00991		

Appendix A – Abstract in Portuguese

O conhecimento sobre a composição da dieta de uma espécie é um passo importante para desvendar a ecologia da mesma e guiar acções de conservação. Isto é especialmente importante para espécies que ocorrem em áreas remotas dentro de *hotspots* de biodiversidade e com pouca informação sobre os papéis ecológicos que desempenham. A emblemática osga-de-muros gigante de Cabo Verde *Tarentola gigas* encontra-se restrita aos ilhéus desabitados de Branco e Raso e apresenta duas subespécies. É classificada como Em Perigo e Extinta localmente na ilha de Santa Luzia, mas pouca informação é conhecida sobre a dieta e comportamento da mesma. Neste estudo, identificámos as principais plantas, artrópodes e vertebrados consumidos por ambas as subespécies da osga usando *NGS* (*metabarcoding* de amostras fecais) e comparámo-las com as espécies conhecidas em Santa Luzia. Os resultados mostram que as plantas têm um papel significativo como itens na dieta e que vertebrados e invertebrados foram identificados com uma resolução taxonómica maior do que com os métodos tradicionais. Com este estudo, temos agora dados sobre a dieta de ambas as subespécies para avaliar a futura reintrodução desta osga ameaçada em Santa Luzia como sendo potencialmente de sucesso, considerando o carácter generalista de ambas as subpopulações. A informação revelada por estas redes ecológicas é importante para o desenvolvimento de planos de conservação pelas autoridades governamentais e reforça o papel essencial e geralmente negligenciado dos répteis em sistemas insulares.
